# Perceptions, Predictors of and Motivation for Quitting among Smokers from Six European Countries from 2016 to 2018: Findings from EUREST-PLUS ITC Europe Surveys

**DOI:** 10.3390/ijerph17176263

**Published:** 2020-08-28

**Authors:** Charis Girvalaki, Filippos T. Filippidis, Christina N. Kyriakos, Pete Driezen, Aleksandra Herbeć, Ute Mons, Sophia Papadakis, Enkeleint A. Mechili, Paraskevi A. Katsaounou, Krzysztof Przewoźniak, Esteve Fernández, Antigona C. Trofor, Tibor Demjén, Geoffrey T. Fong, Constantine I. Vardavas, on behalf of the EUREST-PLUS Consortium

**Affiliations:** 1 Medical School, University of Crete, Heraklion, 71003 Crete, Greece; ckyriakos@tobcontrol.eu (C.N.K.); SPapadakis@ottawaheart.ca (S.P.); mechili@univlora.edu.al (E.A.M.); vardavas@tobcontrol.eu (C.I.V.); 2 European Network for Smoking and Tobacco Prevention, 1050 Brussels, Belgium; 3 Department of Primary Care and Public Health, Imperial College, London W6 8RP, UK; f.filippidis@imperial.ac.uk; 4 Center for Health Services Research, School of Medicine, National and Kapodistrian University of Athens, 10561 Athens, Greece; paraskevikatsaounou@gmail.com; 5 Department of Psychology and School of Public Health and Health Systems, University of Waterloo, Waterloo, ON N2L 3G1, Canada; prdriezen@uwaterloo.ca; 6 Health Promotion Foundation, 00 764 Warsaw, Poland; aaherbec@gmail.com (A.H.); krzysztof.przewozniak@wp.pl (K.P.); geoffrey.fong@uwaterloo.ca (G.T.F.); 7 Centre for Behaviour Change, Clinical, Educational and Health Psychology, University College London, London WC1E 6BT, UK; 8 Cancer Prevention Unit and WHO Collaborating Centre for Tobacco Control, German Cancer Research Center (DKFZ), 69120 Heidelberg, Germany; u.mons@dkfz-heidelberg.de; 9 Heart Center, Faculty of Medicine and University Hospital Cologne, University of Cologne, 50923 Cologne, Germany; 10 Division of Prevention and Rehabilitation, University of Ottawa Heart Institute, and Faculty of Medicine, University of Ottawa, Ottawa, ON K1Y 4W7, Canada; 11 Department of Health Care, Faculty of Public Health, University of Vlora, 9401 Vlora, Albania; 12 First ICU Evaggelismos Hospital Athens, National and Kapodistrian University of Athens, 10676 Athens, Greece; 13 Maria Skłodowska-Curie National Research Institute of Oncology, 02-781 Warsaw, Poland; 14 Collegium Civitas, 00-901 Warsaw, Poland; 15 Tobacco Control Unit, Catalan Institute of Oncology (ICO), 08908 L’Hospitalet de Llobregat, Barcelona, Catalonia, Spain; efernandez@iconcologia.net; 16 Tobacco Control Research Group, Bellvitge Biomedical Research Institute (IDIBELL), 08908 L’Hospitalet de Llobregat, Catalonia, Spain; 17 School of Medicine and Health Sciences, Bellvitge Campus, Universitat de Barcelona, 08908 L’Hospitalet de Llobregat, Catalonia, Spain; 18 Consortium for Biomedical Research in Respiratory Diseases (CIBER of Respiratory Diseases, CIBERES), 28029 Madrid, Spain; 19 University of Medicine and Pharmacy ‘Grigore T. Popa’ Iasi, 700115 Iasi, Romania; atrofor@yahoo.com; 20 Aer Pur Romania, 052034 Bucharest, Romania; 21 Smoking or Health Hungarian Foundation, 1044 Budapest, Hungary; tibor.demjen@gmail.com; 22 Ontario Institute for Cancer Research, Toronto, ON M5G 0A3, Canada

**Keywords:** quit smoking, predictors, reasons, perceptions, European policies, TPD, World Health Organization Framework Convention on Tobacco Control (WHO FCTC)

## Abstract

The European Tobacco Products Directive (TPD) was introduced in 2016 in an effort to decrease prevalence of smoking and increase cessation in the European Union (EU). This study aimed to explore quitting behaviours, motivation, reasons and perceptions about quitting, as well as predictors (reported before the TPD implementation) associated with post-TPD quit status. A cohort study was conducted involving adult smokers from six EU countries (*n* = 3195). Data collection occurred pre-(Wave 1; 2016) and post-(Wave 2; 2018) TPD implementation. Bivariate and logistic regression analyses of weighted data were conducted. Within this cohort sample, 415 (13.0%) respondents reported quitting at Wave 2. Predictors of quitting were moderate or high education, fewer cigarettes smoked per day at baseline, a past quit attempt, lower level of perceived addiction, plans for quitting and the presence of a smoking-related comorbidity. Health concerns, price of cigarettes and being a good example for children were among the most important reasons that predicted being a quitter at Wave 2. Our findings show that the factors influencing decisions about quitting may be shared among European countries. European policy and the revised version of TPD could emphasise these factors through health warnings and/or campaigns and other policies.

## 1. Introduction

Smoking and other forms of tobacco consumption are considered the single most important cause of preventable morbidity and premature mortality worldwide, including in the European Union (EU) [1].

The World Health Organization Framework Convention on Tobacco Control (WHO FCTC) highlights that increasing quit attempts and long-term cessation rates are critical to reducing the prevalence of smoking [2]. WHO FCTC Article 14 calls upon its Parties to take effective measures to promote cessation of tobacco use and adequate treatment for tobacco dependence. WHO FCTC Guidelines for the implementation of Article 14 include a number of evidence-based strategies, such as internet support, quitlines, healthcare professionals’ brief advice, behavioural counselling by trained specialists and use of cessation medications [3].

Research and tobacco dependence treatment guidelines have highlighted the effectiveness of evidence-based tobacco treatment delivery (behavioural counselling and pharmacotherapy) to increase long-term abstinence from smoking [4,5,6], while physicians’ advice and support also play an important role in increasing cessation rates [7,8]. Results from recent studies on the key facilitators of quitting identified, among others, motivation to quit [9], smoking-related health concerns [10,11], concerns about the effects of smoking to others and the money spent on tobacco products [12].

Although the proportion of smokers has declined in most EU countries since 2006, the overall smoking prevalence has remained stable since 2014 [13]. In addition, evidence shows that few European smokers use assistance and evidence-based cessation methods to quit [14]. Greece (37.0%), Hungary (35.0%), Poland (30.0%), Romania (28.0%), Spain (28.0%) and Germany (25.0%) are among the European countries with the highest prevalence of smoking [13]. In these countries, quit ratios have either decreased or remained low even though the population of former smokers has increased in most other European countries in recent years [15].

Efforts to decrease smoking prevalence and increase cessation in the EU also include the European Tobacco Products Directive (TPD) on the manufacture, presentation and sale of tobacco and related products [16]. While the TPD does not have specific provisions for tobacco dependence treatment, many of its measures promote cessation through regulation of tobacco products (e.g., packaging, labelling, ingredients). Although limited data are currently available on the effectiveness of the TPD, previous research has indicated that higher levels of tobacco control policy implementation led to lower prevalence of smoking and higher quit ratios in Europe [15].

Identifying predictors of a successful quit attempt is valuable for tailoring strategies or policies that would help smokers quit, prioritising those most in need of intensive treatment and maximising the use of the healthcare resources available [17]. 

Within the context of the TPD that has changed the tobacco control landscape in the EU, it is also important to understand perceptions of and reasons for quitting as well as study potential changes in quitting behaviour among European smokers during this period. This will improve our understanding of what might help people to quit and inform policies on tobacco control in order to maximise their impact.

In light of the above, using longitudinal data from adult smokers from six EU countries, the purpose of this study was to explore quitting behaviours, motivation to quit, reasons and perceptions about quitting and predictors of smoking cessation within this context.

## 2. Materials and Methods

### 2.1. Design and Participants

The current study is part of the Horizon 2020 funded project, European Regulatory Science on Tobacco: Policy implementation to reduce lung diseases (EUREST_PLUS) [18]. Data come from the International Tobacco Control Policy Evaluation (ITC) Project [19] Six European Country (6E) Survey, a cohort study using a pre vs. post-TPD implementation design in six EU member states (MS)—Germany, Greece, Hungary, Poland, Romania and Spain. A probabilistic, stratified cluster sampling design was used to randomly sample respondents within geographic strata that were defined according to the Nomenclature of Territorial Units for Statistics (NUTS) regions and degree of urbanisation (urban, intermediate, rural). Clusters, approximately the size of enumeration areas, were randomly selected proportional to population size within strata. Households were selected within clusters using a random walk method and up to two smokers (one male, one female, where possible) were randomly selected within households. Interviews were conducted face-to-face using computer assisted personal interviews (CAPI). Further details about the study methodology are provided elsewhere [20,21,22,23].

The Wave 1 (June–September 2016) sample included 6011 adults (≥18 years) who reported smoking cigarettes at least monthly and smoked 100 or more cigarettes in their lifetime. In Wave 2 (February–May 2018), 53% of respondents were retained at follow up (*n* = 3195). Demographic differences between Waves 1 and 2 due to attrition were generally small (Supplementary Table S1).

### 2.2. Demographic Characteristics

Demographic characteristics examined were age (18–24, 25–39, 40–54 and ≥55 years); gender (male, female); degree of urbanisation (urban, intermediate, rural); highest level of formal education completed (low, moderate, and high); monthly gross household income (low, moderate and high) [20,24].

### 2.3. Smoking Behaviours

Current smoking status was categorised as either daily smoker or non-daily smoker (less than daily but at least once per month). In Wave 2, respondents were considered to have quit smoking if they reported not smoking for at least 30 days at the time of follow up. Smokers were asked how many cigarettes they smoked per day (<10; 11–20; 21–30; 31+) and whether they smoked exclusively FM (factory-made) cigarettes, RYO (roll-your-own) tobacco or both FM and RYO. Nicotine dependence was assessed using the Heaviness of Smoking Index (low (0–1), moderate (2–4), high (5–6)) [25] and perceived addiction with response options “not at all addicted”, “somewhat addicted” and “very addicted”.

### 2.4. Quitting Behaviours

Attempts and intentions to quit smoking were examined with the questions, “Have you ever tried to quit smoking?” (yes, no), “How many quit attempts have you made in the last 12 months?” (no attempt, 1+), “Are you planning to quit smoking…?” (within the next month, the next 6 months, beyond 6 months, no plans to quit), “Have you made an attempt to quit smoking in the last 12 months?” (yes, no) and “How much do you want to quit smoking?” (not at all, a little, somewhat, a lot).

### 2.5. Perceptions and Reasons for Quitting

Perceptions around smoking were assessed with the questions, “Ιn the last 30 days how often did you think about: the harm your smoking might be doing to you?, the harm your smoking might be doing to other people?, the money you spend on smoking?” (never, rarely, sometimes, often, very often); “In the last 30 days, have you stubbed out a cigarette before you finished it because you thought about the harm of smoking?” (yes, no) and “How much do you think you would benefit from health and other gains if you were to quit smoking permanently in the next 6 months?” (not at all, slightly, moderately, very much, extremely).

Respondents were asked, “If you decided to give up smoking completely in the next 6 months, how sure are you that you would succeed?” (not at all sure, slightly, moderately, very, extremely) and “How difficult would it be for you to quit smoking if you wanted to?” (not at all, slightly, moderately, very, extremely).

Respondents were asked about reasons for quitting: “In the past 6 months, how much have each of the following things led you to think about quitting…concern for your personal health?, concern for the effect of your cigarette smoke on non-smokers?, that society disapproves of smoking?, the price of cigarettes?, smoking restrictions at work?, smoking restrictions at public places like restaurants, cafes and pubs?, availability of telephone helpline/quitline/information line?, advice from a health professional to quit?, free or lower cost, stop-smoking medication?, warning labels on cigarette packages?, setting an example for children?, close friends and family disapprove of your smoking?, a prevention message or campaign?, being told you had a smoking related illness?, planning to have or expecting a child?” (not at all, somewhat, very much).

### 2.6. Comorbidities and Depressive Symptoms

The presence of comorbidities was assessed with the questions, “Are you currently being treated for, or do you have a current diagnosis for, any of the following?: chronic pain?, diabetes?, cancer?, heart disease?, chronic lung disease (COPD, emphysema or chronic bronchitis)?, asthma?, tuberculosis?” (yes, no).

Respondents were classified as displaying depressive symptoms [26] if they responded yes to at least one of the following three questions: “During the last 30 days, have you often been bothered by little interest or pleasure in doing things?”, “During the last 30 days, have you often been bothered by feeling down, depressed, or hopeless?” and “Are you currently being treated for, or do you have a current diagnosis for depression?” (yes/no).

### 2.7. Statistical Analysis

Descriptive data are shown as weighted percentages and 95% confidence intervals (CI). Bivariate analysis examined the association between quit status at Wave 2 and smoking behaviours, quitting behaviours, cessation assistance, perceptions and reasons for quitting, comorbidities and depressive symptoms at Wave 1 (chi square test). A logistic regression model estimated the odds of having quit by Wave 2 as a function of the following factors measured at Wave 1: daily cigarette consumption, number of quit attempts, perceived addiction, plans to quit, self-efficacy, comorbidities and a positive screen for depression. This regression model controlled for country, gender, age group, degree of urbanization, household income and education. All statistical tests and CIs accounted for the complex sampling design, with sampling region as strata and primary sampling unit as clusters. All *p*-values reported were two-tailed. Bivariate and logistic regression analyses of weighted data was conducted using SAS-callable SUDAAN (Version 11.0.3).

## 3. Results

### 3.1. Quitters’ Demographic Characteristics

The analytic sample consisted of 3195 adult cigarette smokers recruited at Wave 1 and successfully followed up at Wave 2. Within this cohort sample, 415 (13.0%) respondents reported having quit at Wave 2. Specifically, 11.5% of the male and 14.0% of the female respondents at Wave 1 had quit smoking at Wave 2. Having quit smoking at Wave 2 was reported by 16.2% of respondents in Spain, 14.8% in Romania, 13.8% in Greece, 12.0% in Hungary, 10.2% in Poland and 8.3% in Germany. Finally, 13.8% of the residents of urban locations, 13.2% of those with moderate household income and 20.7% of those with high education at Wave 1 were quitters at Wave 2 (Supplementary Table S1). Continuing smokers had similar demographic characteristics as quitters.

### 3.2. Wave 1 Smoking Behaviours and Quitting in Wave 2

As presented in Table 1, 30.0% of non-daily smokers at Wave 1 quit by Wave 2 compared to 11.5% of daily smokers (*p* < 0.001). Quit rates were higher among FM smokers compared to those using RYO or both (*p* = 0.004) and among those smoking <10 cigarettes per day at Wave 1 (*p* < 0.001). Among those with low HSI and those who did not perceive themselves as addicted at Wave 1, 17.5% and 21.2% quit smoking by Wave 2, respectively (*p* < 0.001). In addition, 14.2% of the quitters had tried to quit in the past and 20.1% had done so within the previous year compared to 10.4% (*p* = 0.005) and 10.9% (*p* < 0.001) among non-quitters, respectively. Among those who intended to quit within the next month and desired a lot to quit at Wave 1, 26.9% and 18.4%, respectively, succeeded by Wave 2. 

### 3.3. Wave 1 Perceptions about Smoking and Quitting in Wave 2

Table 2 presents respondents’ perceptions about smoking by smoking status at Wave 2. Both quitters and continuing smokers reported thinking about the harm of smoking to themselves sometimes (32.0% vs. 30.7%) or often (20.9% vs. 19.3%), about the harm of smoking to others sometimes (29.8% vs. 28.3%) or never (24.6% vs. 25.2%) and about money spent on smoking sometimes (28.2% vs. 25.1%).

Table 2 also depicts reasons for quitting at Wave 1 by smoking status at Wave 2. A greater percentage of those who had quit smoking by Wave 2 reported being very/extremely sure that they could quit at Wave 1 compared to continuing smokers (25.8% vs. 13.9%, respectively; *p* < 0.001). Continuing smokers had also perceived quitting as something very/extremely difficult at Wave 1 compared to quitters (49.0% vs. 33.7%, *p* < 0.001).

Concerns for personal health, price of cigarettes, being a good example for children and having a smoking related illness are among the reasons which were considered as the most important for quitting at Wave 1, with little difference according to quit status at Wave 2. Reasons such as the effect on non-smokers’ health, advice from a healthcare professional and friends and family’s disapproval were considered as somewhat important reasons for quitting for almost a quarter of respondents. However, reasons such as societal disapproval, restrictions at work and at public places, helpline availability, affordability of stop-smoking medications, labels on cigarette packages, prevention messages/campaigns and plans to have a child were considered not at all important. Finally, only around one in ten respondents had indicated that they would not benefit at all if they quit smoking, with no statistically significant difference when contrasted with quit status at Wave 2.

### 3.4. Factors Associated with Being a Quitter during Wave 2

Table 3 presents the Wave 1 factors associated with having quit smoking by Wave 2. Living in Greece (odds ratio (OR) 2.35; 95% CI 1.23–4.48), Hungary (OR 2.16; 95% CI 1.12–4.17) or Spain (OR 2.41; 95% CI 1.29–4.49) was associated with higher odds of quitting smoking during Wave 2 compared to living in Germany. Respondents with moderate (OR 1.46; 95% CI 1.05–2.03) or high education (OR 2.16; 95% CI 1.42–3.29) were more likely to be quitters during Wave 2 than those with low education. Daily cigarette consumption (11–20 cigarettes/day vs. ≤10 cigarettes/day: OR 0.64; 95% CI 0.47–0.86; 21+ cigarettes/day vs. ≤10 cigarettes/day: OR 0.30; 95% CI 0.18–0.50) was negatively associated with quitting smoking during Wave 2 while at least one past quit attempt was positively associated with being a quitter (OR 1.58; 95% CI 1.15–2.18). Lower level of perceived addiction (OR 2.04; 95% CI 1.34–3.11) as well as making plans for quitting within the next month (OR 1.90; 95% CI 1.13–3.20) were also associated with higher odds of being a quitter in Wave 2. Finally, the presence of a smoking related comorbidity predicted quitting (OR 1.59; 95% CI 1.07–2.35).

## 4. Discussion

This is the first cohort study of adult smokers from multiple EU countries exploring quitting behaviours, motivation to quit, reasons and perceptions about quitting and implemented policies as well as factors (reported before TPD implementation) associated with post-TPD quit status. Moderate or high education, consumption of <10 cigarettes per day, lower perceived addiction, at least one past quit attempt, planning to quit and the presence of a smoking related comorbidity were identified as predictors of being a quitter at Wave 2. Among reasons to quit smoking, policy aspects such as restrictions at work, restrictions at public places, labels on cigarette packages and prevention messages/campaigns as well as helpline availability and affordability of stop-smoking medications were generally not considered as important. However, perceived harm of smoking to their health or others, being a good example for children, having a smoking related illness and money spent on smoking were reported as important factors for quitting.

Consistent with our findings, previous studies have shown that the level of confidence in succeeding to quit is an important factor, also indicative of the success [27,28]. Quitters at Wave 2 also planned to quit at Wave 1, although levels of desire to quit were low. The existing evidence supports the role of motivation and desire in smoking cessation. However, these factors may be predictors of attempting to quit but not of maintaining abstinence [29,30,31,32]. This indicates that motivation may be important as a first step in the quit attempt process, but other factors could contribute to quit success. How different aspects of the motivational system influence behaviour and maintenance should be investigated in future research. Healthcare professionals’ support in boosting patients’ confidence in successful quitting could be incorporated into clinical practice along with information on the efficacy of pharmacotherapy and behavioural advice and enhance smokers’ chances of succeeding.

Our analysis also showed that higher education and household income are associated with quitting, which aligns with previous research [33,34,35]. In addition, our findings concerning lighter smoking and low levels of perceived addiction among those who quit [36,37,38] as well as the presence of a disease [10,27,39] or health concerns about smoking [10,40,41] are consistent with the literature. Others have found that suffering from a chronic disease or multiple comorbidities caused or worsened by smoking do not always lead to cessation [42], which was true for some of our study’s respondents. This may be explained by the fact that a successful attempt is associated with the level of nicotine dependence [43], which is often higher in smokers with chronic diseases [44,45] and with the intensity of the medical support for smoking cessation provided [45]. 

Money spent on smoking was generally reported as an important reason for quitting, which again highlights that price increases and taxes on tobacco products are very effective tobacco control measures [46,47]. This might partly explain why somewhat more FM cigarette smokers quit between Waves 1 and 2 compared to RYO and users of both. Recent increases in FM cigarette prices in Europe have been associated with increased use of RYO, which is a cheaper form of tobacco [48,49,50].

Helpline availability and affordable stop-smoking medications were also not perceived as important reasons for quitting. These are both effective strategies to support cessation but smokers may be unaware of the effectiveness of smoking cessation treatment, underestimate their benefits [51] or hesitate to use such services, as in many European countries, they are not reimbursed [52,53]. As a result, use of evidence-based smoking cessation treatment and cessation aids in general is suboptimal in Europe [13,54,55,56]. Increased funding for smoking cessation services and aids especially for high risk populations (e.g., those with chronic diseases, severe mental illness, addiction HIV, Tuberculosis, as well as pregnant women) and building on the capacity of healthcare professionals to provide tobacco treatment services, (e.g., by offering hands-on training or digital training [57,58]) could improve the smoking cessation landscape in Europe. 

While not reported as key factors for quitting among the respondents in our study, there is clear evidence that tobacco control policies, such as smoking restrictions at work or in public places and warning labels on cigarette packages, contribute to smoking cessation and reduce smoking prevalence [10,14,47]. Romania, Poland, Spain and Germany scored low or dropped on the Tobacco Control Scale ranking since 2016 [59], while the TPD had not been fully implemented in all EU countries by 2016, [60] when we conducted Wave 1 data collection. For example, only Hungary, Romania and Spain had pictorial warnings on the back side of the packages and the implementation of smoke-free legislation was poor in Greece, Poland and Germany [61]. Thus, the perceptions regarding smoking restrictions at work or in public places may simply reflect relatively poor implementation at the time of data collection in the countries assessed. Such differences between countries may partly explain the variation in the proportion of smokers who reported quitting between Wave 1 and Wave 2. Greece, for instance, implemented new tobacco control measures during the study period, improving its position in the Tobacco Control Scale, whereas Poland dropped from the 15th to the 23rd place in the same scale from 2016 to 2019 [59,61].

Overall, our findings regarding the reasons and predictors of quitting in Europe such as concerns for personal health [10,62], price of cigarettes, smoking related illness, advice from a healthcare professional, family disapproval, [10], being a good example for their children [63] and the effects of smoking on others [11] are aligned with previous European studies. This shows that reasons influencing decisions about quitting may be shared among European countries. European policy and the revised version of TPD could emphasise the factors related to the health risks of smoking and benefits of smoking cessation for oneself and others which seem to influence smokers decisions—for instance, through health warnings and/or campaigns and other policies along with other characteristics such as flavours, which have been found to influence smoking and cessation characteristics of the users [64].

### Study Strengths and Limitations

Although results regarding quitting and cessation behaviours from both waves of the EUREST-PLUS Europe Surveys have been published [52,64,65,66], this study is the first to present cohort data on predictors, motivation, reasons and perceptions for quitting. In addition, this study examined an extensive number of measures and used validated tools. However, the comparably high rate of smokers who were lost to follow up (45%) is an important limitation as differential attrition may have introduced bias. While there were some slight differences between retained vs. lost respondents, these differences were generally small. For instance, given that we did not find significant differences in intentions to quit smoking at Wave 1 (retained vs. lost respondents), associations with successful cessation by Wave 2 might not be biased [67]. Additionally, our analysis was based on self-reported data, which may be subject to information bias (e.g., social desirability bias). We assessed multiple variables and thus conducted multiple comparisons. Although this carries the risk of finding statistically significant differences by chance, most of the differences detected were significant at the 0.001 level, which increases our confidence in the findings. Perceptions, reasons and motivation may also change over time. Given the fact that we assessed Wave 1 in 2016, some respondents may have lost or found motivation, perceptions and reasons to quit may have changed, with the implementation of TPD measures possibly having contributed to this change. In addition, there may be differences between countries in how people respond to questions about perceptions and motivation. Finally, quitting was self-reported and defined as 30-day abstinence; we cannot verify that respondents remained abstinent from smoking.

## 5. Conclusions

This study is the first to present results from the largest cross-national cohort sample of smokers in Europe, examining the predictors of quitting, motivation, reasons and perceptions about quitting among smokers from multiple EU MS, from 2016 to 2018. Perceptions of the personal and social impact of smoking were considered more important than typical tobacco control policies at the time of the study when the European landscape of tobacco control policies was changing. Although perceptions do not necessarily reflect the actual effectiveness of various policies, these findings can inform future tobacco control policies in Europe—for example, when designing warning labels or public campaigns. They can also be incorporated into the revision of the TPD to enhance and strengthen existing policies that appeal to smokers’ concerns and motivations to quit.

## Figures and Tables

**Table 1:** Smoking behaviour and doctors visit characteristics at Wave 1 among smokers who quit by Wave 2 (2018) (*n* = 3195) of EUREST-PLUS ITC 6 European Country Survey.

Wave 1 Smoking Behaviours	(N Quit)	% Quit		*p*-Value **
		%	(95% CI)	
Smoking status				
Daily smoker	(369/3020)	11.5	(9.9, 13.3)	**<0.001**
Non-daily smoker	(46/175)	31.0	(21.9, 41.2)	
Smokes FM/RYO *				
FM only	(330/2373)	13.9	(11.9, 16.2)	**0.004**
RYO only	(59/535)	10.3	(7.8, 13.5)	
Both	(26/287)	7.2	(4.2, 11.5)	
Cigarettes/day				
<10	(219/1154)	20.2	(17.0, 23.7)	**<0.001**
11–20	(168/1576)	10.1	(8.3, 12.1)	
21–30	(16/297)	3.5	(1.7, 6.3)	
31+	(12/163)	5.3	(2.3, 10.3)	
Heaviness of Smoking Index				
Low (0–1)	(125/703)	17.5	(14.0, 21.5)	**<0.001**
Moderate (2–4)	(229/2035)	10.6	(9.0, 12.5)	
High (5–6)	(15/272)	4.1	(2.0, 7.1)	
Perceived addiction				
Not at all	(90/410)	21.2	(16.7, 26.5)	**<0.001**
Somewhat	(210/1491)	14.4	(12.1, 17.1)	
Very addicted	(115/1287)	7.4	(5.8, 9.3)	
Ever tried to quit smoking				
Never tried to quit	(143/1344)	10.4	(8.4, 12.9)	**0.005**
Ever tried to quit	(272/1851)	14.2	(12.2, 16.5)	
Intentions to quit				
In the next month	(42/142)	26.9	(18.2, 37.1)	**<0.001**
In the next 6 months	(50/268)	16.8	(11.5, 23.3)	
Beyond 6 months	(131/990)	14.0	(11.4, 16.9)	
No plans to quit	(192/1795)	10.2	(8.3, 12.5)	
Desire to quit				
Not planning to quit	(192/1795)	10.2	(8.3, 12.5)	**<0.001**
A little	(32/300)	11.3	(6.8, 17.2)	
Somewhat	(85/573)	15.7	(12.3, 19.7)	
A lot	(105/522)	18.4	(14.5, 22.9)	
Attempts to quit in past year				
Did not try	(291/2586)	10.9	(9.2, 12.8)	**<0.001**
Tried	(124/609)	20.1	(16.2, 24.7)	

* FM = factory-made cigarettes; RYO = roll-your-own tobacco. ** Chi square test. **Bold** = *p* < 0.05.

**Table 2:** Perceptions and reasons for quitting of the cohort sample (*n* = 3195) of EUREST-PLUS ITC 6E Survey at Wave 1 (2016) and quit status at Wave 2 (2018).

Perceptions and Reasons for Quitting	Quit Status in Wave 2						*p*-Value *
	Quitters = 415			Continuing Smokers = 2780			
	*n*	%	(95% CI)	*n*	%	(95% CI)	
Wave 1 Perceptions							
Think about harm of smoking							
Never	80	21.3	(16.3, 27.3)	590	21.8	(19.5, 24.4)	0.917
Rarely	75	17.7	(13.5, 22.8)	527	19.4	(17.3, 21.7)	
Sometimes	127	32.0	(26.6, 37.9)	863	30.7	(28.3, 33.2)	
Often	94	20.9	(16.5, 26.0)	544	19.3	(17.3, 21.4)	
Very often	39	8.2	(5.2, 12.0)	249	8.8	(7.4, 10.4)	
Think about harm to others							
Never	92	24.6	(18.8, 31.4)	684	25.2	(22.5, 28.1)	0.965
Rarely	86	22.5	(17.8, 28.0)	584	22.2	(20.2, 24.4)	
Sometimes	130	29.8	(24.9, 35.3)	814	28.3	(25.8, 31.0)	
Often	72	15.9	(12.1, 20.7)	485	17.3	(15.4, 19.3)	
Very often	34	7.1	(4.5, 10.6)	207	7.0	(5.7, 8.5)	
Think about money spent on smoking							
Never	87	23.1	(18.3, 28.8)	462	16.9	(14.6, 19.4)	0.058
Rarely	54	12.3	(9.0, 16.5)	348	13.7	(11.9, 15.8)	
Sometimes	114	28.2	(23.1, 33.9)	717	25.1	(22.9, 27.5)	
Often	85	19.4	(14.9, 24.9)	696	25.7	(23.5, 28.1)	
Very often	74	17.0	(12.8, 22.3)	554	18.5	(16.4, 20.9)	
Stubbed out cigarette before finishing							
Yes	70	16.2	(12.2, 21.1)	444	16.2	(13.9, 18.8)	0.982
No	342	83.8	(78.9, 87.8)	2335	83.8	(81.2, 86.1)	
Wave 1 reasons for quitting							
Confidence to quit							
Not at all sure	90	22.4	(17.6, 28.2)	932	33.6	(31.1, 36.3)	**<0.001**
Slightly sure	108	27.3	(22.2, 32.9)	831	30.5	(27.9, 33.2)	
Moderately sure	96	24.5	(19.8, 29.8)	576	22.0	(19.9, 24.3)	
Very sure	65	16.3	(12.7, 20.7)	235	8.1	(6.9, 9.4)	
Extremely sure	40	9.5	(6.0, 14.2)	144	5.8	(4.6, 7.2)	
Difficulty in quitting							
Not at all difficult	53	14.1	(10.4, 19.0)	174	6.9	(5.6, 8.4)	**<0.001**
Slightly difficult	99	24.6	(20.6, 29.1)	473	17.9	(16.0, 20.0)	
Moderately difficult	113	27.6	(22.2, 33.7)	738	26.3	(24.0, 28.7)	
Very difficult	94	22.7	(18.1, 28.1)	838	30.1	(27.7, 32.5)	
Extremely difficult	53	11.0	(8.2, 14.6)	533	18.9	(16.9, 21.1)	
Reasons for quitting: concern for personal health							
Not at all	94	25.4	(19.7, 32.2)	701	27.1	(24.6, 29.8)	0.188
Somewhat	177	42.3	(36.4, 48.5)	1292	46.3	(43.3, 49.3)	
Very much	143	32.3	(26.5, 38.7)	762	26.6	(24.2, 29.1)	
Reasons for quitting: effect on non-smokers health							
Not at all	174	43.7	(37.1, 50.6)	1178	44.5	(41.5, 47.4)	0.754
Somewhat	167	39.1	(33.2, 45.3)	1142	40.0	(37.3, 42.8)	
Very much	74	17.2	(13.3, 21.8)	435	15.5	(13.6, 17.7)	
Reasons for quitting: society disapproves							
Not at all	262	67.3	(61.0, 73.0)	1889	70.1	(67.0, 73.0)	0.651
Somewhat	106	24.7	(19.8, 30.5)	656	23.1	(20.6, 25.7)	
Very much	38	7.9	(5.0, 11.9)	186	6.9	(5.7, 8.2)	
Reasons for quitting: price of cigarettes							
Not at all	115	29.7	(23.6, 36.7)	781	30.3	(27.6, 33.2)	0.976
Somewhat	153	37.7	(31.7, 44.1)	1045	37.6	(34.9, 40.3)	
Very much	147	32.6	(27.1, 38.7)	935	32.1	(29.2, 35.0)	
Reasons for quitting: restrictions at work							
Not at all	277	67.6	(61.1, 73.6)	1892	69.8	(66.7, 72.7)	0.779
Somewhat	93	23.5	(18.3, 29.7)	598	21.6	(19.3, 24.2)	
Very much	40	8.8	(5.7, 13.0)	211	8.6	(7.1, 10.3)	
Reasons for quitting: restrictions in public places							
Not at all	265	64.8	(58.2, 70.8)	1819	66.1	(62.7, 69.3)	0.727
Somewhat	107	26.7	(21.4, 32.8)	699	24.6	(21.8, 27.6)	
Very much	41	8.5	(5.4, 12.5)	238	9.4	(7.8, 11.2)	
Reasons for quitting: telephone helpline available							
Not at all	319	80.5	(74.8, 85.2)	2194	80.5	(77.7, 83.1)	0.998
Somewhat	68	15.2	(11.1, 20.6)	408	15.2	(13.0, 17.7)	
Very much	19	4.2	(2.0, 7.7)	117	4.3	(3.3, 5.5)	
Reasons for quitting: advice from health professional							
Not at all	230	59.1	(52.6, 65.2)	1694	62.7	(59.6, 65.6)	0.253
Somewhat	132	30.9	(25.6, 36.8)	734	26.5	(24.0, 29.0)	
Very much	48	10.0	(6.6, 14.3)	310	10.9	(9.4, 12.6)	
Reasons for quitting: affordable stop-smoking medications							
Not at all	297	78.0	(72.7, 82.5)	1895	71.6	(68.7, 74.4)	**0.033**
Somewhat	60	13.1	(9.8, 17.2)	460	18.3	(16.0, 20.9)	
Very much	38	8.9	(6.0, 12.8)	285	10.0	(8.6, 11.6)	
Reasons for quitting: labels on cigarette packages							
Not at all	265	67.5	(61.0, 73.4)	1926	70.3	(67.2, 73.3)	0.348
Somewhat	106	23.8	(19.0, 29.4)	648	23.6	(20.9, 26.5)	
Very much	42	8.7	(5.6, 12.7)	174	6.1	(4.9, 7.6)	
Reasons for quitting: example for children							
Not at all	171	42.6	(36.1, 49.4)	1150	43.5	(40.4, 46.6)	0.710
Somewhat	126	31.3	(25.7, 37.5)	913	32.7	(30.0, 35.6)	
Very much	116	26.1	(20.9, 32.0)	660	23.8	(21.5, 26.3)	
Reasons for quitting: friends and family disapprove							
Not at all	228	59.0	(52.4, 65.3)	1710	62.6	(59.4, 65.6)	0.505
Somewhat	130	29.3	(23.6, 35.6)	757	27.1	(24.6, 29.7)	
Very much	56	11.7	(8.9, 15.4)	273	10.4	(8.9, 12.1)	
Reasons for quitting: prevention message campaign							
Not at all	296	75.2	(69.2, 80.4)	2044	74.4	(71.3, 77.2)	0.873
Somewhat	90	19.8	(15.1, 25.6)	535	20.0	(17.6, 22.7)	
Very much	27	5.0	(3.1, 7.6)	153	5.6	(4.4, 7.1)	
Reasons for quitting: have smoking related illness							
Not at all	221	54.4	(47.7, 60.9)	1499	56.8	(53.6, 60.0)	0.677
Somewhat	97	23.2	(18.3, 28.9)	650	22.9	(20.4, 25.5)	
Very much	96	22.5	(18.1, 27.6)	569	20.3	(18.0, 22.8)	
Reasons for quitting: planning to have a child							
Not at all	277	67.0	(61.1, 72.5)	1911	71.1	(68.2, 73.9)	0.334
Somewhat	73	17.7	(13.5, 22.9)	418	16.7	(14.4, 19.2)	
Very much	58	15.2	(11.4, 20.2)	324	12.2	(10.5, 14.2)	
How much benefit if you quit smoking							
Not at all	29	8.3	(5.1, 12.8)	272	10.6	(9.0, 12.6)	0.535
Slightly	73	17.4	(13.3, 22.5)	515	19.3	(17.2, 21.7)	
Moderately	120	30.9	(25.0, 37.5)	857	31.3	(28.7, 33.9)	
Very much	135	30.4	(24.9, 36.5)	782	28.4	(25.8, 31.2)	
Extremely	47	13.0	(8.9, 18.1)	285	10.3	(8.6, 12.4)	

* Chi square test.**Bold** = *p* < 0.05. Wave 1 Reasons for Quitting and Status in Wave 2.

**Table 3:** Wave 1 factors associated with being a quitter at Wave 2 of the cohort sample (*n* = 3100) of EUREST-PLUS ITC 6E Surveys.

	Quitter at Wave 2			
Effect	OR	95% CI		*p*-Value
Country				
Germany	1.00			
Greece	2.35	(1.23, 4.48)		**0.010**
Hungary	2.16	(1.12, 4.17)		**0.021**
Poland	1.29	(0.69, 2.44)		0.427
Romania	1.89	(0.99, 3.60)		0.055
Spain	2.41	(1.29, 4.49)		**0.006**
Gender				
Male	1.00			
Female	1.08	(0.86, 1.37)		0.491
Age group				
18–24	1.00			
25–39	0.89	(0.54, 1.45)		0.642
40–54	1.03	(0.61, 1.75)		0.901
55+	1.11	(0.67, 1.82)		0.683
Area of living				
Urban	1.00			
Intermediate	0.89	(0.63, 1.26)		0.512
Rural	0.98	(0.62, 1.55)		0.925
Household income				
Low	1.00			
Moderate	1.19	(0.80, 1.78)		0.381
High	1.06	(0.65, 1.72)		0.822
Not reported	1.06	(0.64,1.75)		0.817
Level of education				
Low	1.00			
Moderate	1.46	(1.05, 2.03)		**0.024**
High	2.16	(1.42, 3.29)		**<0.001**
Cigarettes per day				
≤10	1.00			
11–20	0.64	(0.47, 0.86)		**0.003**
21+	0.30	(0.18, 0.50)		**<0.001**
Number of quit attempts				
No attempts to quit	1.00			
At least one attempt	1.58	(1.15, 2.18)		**0.005**
Perceived addiction level				
Very addicted	1.00			
Somewhat addicted	1.63	(1.18, 2.25)		**0.003**
Not at all	2.04	(1.34, 3.11)		**0.001**
Plans to quit				
No plans to quit	1.00			
Sometime in the future	1.28	(0.94, 1.72)		0.111
In the next month	1.90	(1.13, 3.20)		**0.016**
Self-efficacy				
Not at all/slightly/moderately sure	1.00			
Very/extremely sure can quit	1.36	(0.99, 1.87)		0.058
Comorbidities *				
No	1.00			
Yes	1.59	(1.07, 2.35)		**0.021**
Positive screen for depression				
No	1.00			
Yes	0.88	(0.63, 1.23)		0.455

* Comorbidities = Are you currently being treated for or do you have a current diagnosis for any of the following? (chronic pain, diabetes, cancer, heart disease, chronic lung disease (COPD, emphysema or chronic bronchitis), asthma, tuberculosis). **Bold** = *p* < 0.05.

## Supplementary Materials

The following are available online at https://www.mdpi.com/1660-4601/17/17/6263/s1, Table S1: Demographic characteristics at Wave 1 among smokers who were quit by Wave 2 (2018), (*n* = 3195), of EUREST-PLUS ITC 6E Survey.
