# Supplementary material for: Perceptions, Predictors of and Motivation for Quitting among Smokers from Six European Countries from 2016 to 2018: Findings from EUREST-PLUS ITC Europe Surveys
Source: Int J Environ Res Public Health. 2020 Aug 28;17(17):6263. doi: 10.3390/ijerph17176263 (PMC7504326; doi:10.3390/ijerph17176263)
Supplement: Supplementary file 1 [file ijerph-17-06263-s001.docx]

**Supplementary Table S1:** **Demographic characteristics at Wave 1 among smokers who were quit by Wave 2 (2018), (n = 3195), of EUREST-PLUS ITC 6E Survey.**

| **Wave 1 Demographics** | **% Quit** | | |  |
| --- | --- | --- | --- | --- |
|  |  | | |  |
|  | **(N Quit)** | **%** | **(95% CI)** | **p-Value *** |
| **Country** | | | |  |
| Germany | (62/707) | 8.3 | (5.4, 12.7) | 0.062 |
| Greece | (59/413) | 13.8 | (10.1, 18.5) |  |
| Hungary | (48/357) | 12.0 | (7.8, 17.4) |  |
| Poland | (46/459) | 10.2 | (7.2, 13.9) |  |
| Romania | (82/545) | 14.8 | (11.0, 19.6) |  |
| Spain | (118/714) | 16.2 | (12.2, 21.2) |  |
| **Residential location** | | | |  |
| Urban | (176/1230) | 13.8 | (11.4, 16.6) | 0.245 |
| Intermediate | (136/1171) | 10.8 | (8.5, 13.6) |  |
| Rural | (103/794) | 12.8 | (9.3, 17.4) |  |
| **Gender** | | | |  |
| Male | (212/1615) | 11.5 | (9.6, 13.8) | 0.059 |
| Female | (203/1580) | 14.0 | (11.9, 16.4) |  |
| **Age group** | | | |  |
| 18-24 | (39/244) | 13.7 | (8.9, 19.7) | 0.917 |
| 25-39 | (107/888) | 12.0 | (9.7, 14.7) |  |
| 40-54 | (140/1111) | 12.8 | (10.1, 16.0) |  |
| 55+ | (129/952) | 12.7 | (10.3, 15.6) |  |
| **Household income** | | | |  |
| Not reported | (103/696) | 14.4 | (11.2, 18.2) | 0.276 |
| Low | (84/786) | 10.2 | (7.4, 13.8) |  |
| Moderate | (162/1190) | 13.2 | (10.8, 16.0) |  |
| High | (66/523) | 12.0 | (8.6, 16.3) |  |
| **Education** | | | |  |
| Low | (129/1232) | 9.5 | (7.3, 12.3) | **<0.001** |
| Moderate | (225/1625) | 13.3 | (11.3, 15.5) |  |
| High | (60/326) | 20.7 | (15.6, 27.0) |  |

* Chi square test. **Bold**=p < 0.05
